# Crystal structure of di­aqua­bis­(2,6-di­methyl­pyrazine-κ*N* ^4^)bis­(thio­cyanato-κ*N*)cobalt(II) 2,5-di­methyl­pyrazine monosolvate

**DOI:** 10.1107/S2056989015021829

**Published:** 2015-12-06

**Authors:** Stefan Suckert, Susanne Wöhlert, Inke Jess, Christian Näther

**Affiliations:** a Institut für Anorganische Chemie, Christian-Albrechts-Universität Kiel, Max-Eyth-Strasse 2, 24118 Kiel, Germany

**Keywords:** crystal structure, coordination polymer, octa­hedral coordination, cobalt(II)

## Abstract

In the crystal structure of the title compound, [Co(NCS)_2_(C_6_H_8_N_2_)_2_(H_2_O)_2_]·C_6_H_8_N_2_, the Co^II^ cation is coordinated by the N atoms of two terminal thio­cyanate anions, the O atoms of two water mol­ecules and two N atoms of two 2,6-di­methyl­pyrazine ligands. The coordination sphere of the resulting discrete complex is that of a slightly distorted octa­hedron. The asymmetric unit comprises a Co^II^ cation and half of a 2,5-di­methyl­pyrazine ligand, both of which are located on centres of inversion, and a water ligand, a 2,6-di­­methyl­pyrazine ligand and one thio­cyanate anion in general positions. In the crystal, the discrete complexes are arranged in such a way that cavities are formed in which the 2,5-di­methyl­pyrazine solvent mol­ecules are located. The coordination of the 2,5-di­methyl­pyrazine mol­ecules to the metal is apparently hindered due to the bulky methyl groups in vicinal positions to the N atoms, leading to a preferential coordination of the 2,6-di­methyl­pyrazine ligands. The discrete complexes are linked by O—H⋯N hydrogen bonds between one water H atom and the non-coordinating N atom of the 2,6-di­methyl­pyrazine ligands. The remaining water H atom is hydrogen bonded to one N atom of the 2,5-di­methyl­pyrazine solvent mol­ecule. This arrangement leads to the formation of a two-dimensional network extending parallel to (010).

## Related literature   

For structures with metal thio­cyanates and 2,5-di­methyl­pyrazine or 2,6-di­methyl­pyrazine, see: Otieno *et al.* (2003 ▸); Mahmoudi & Morsali (2009 ▸).
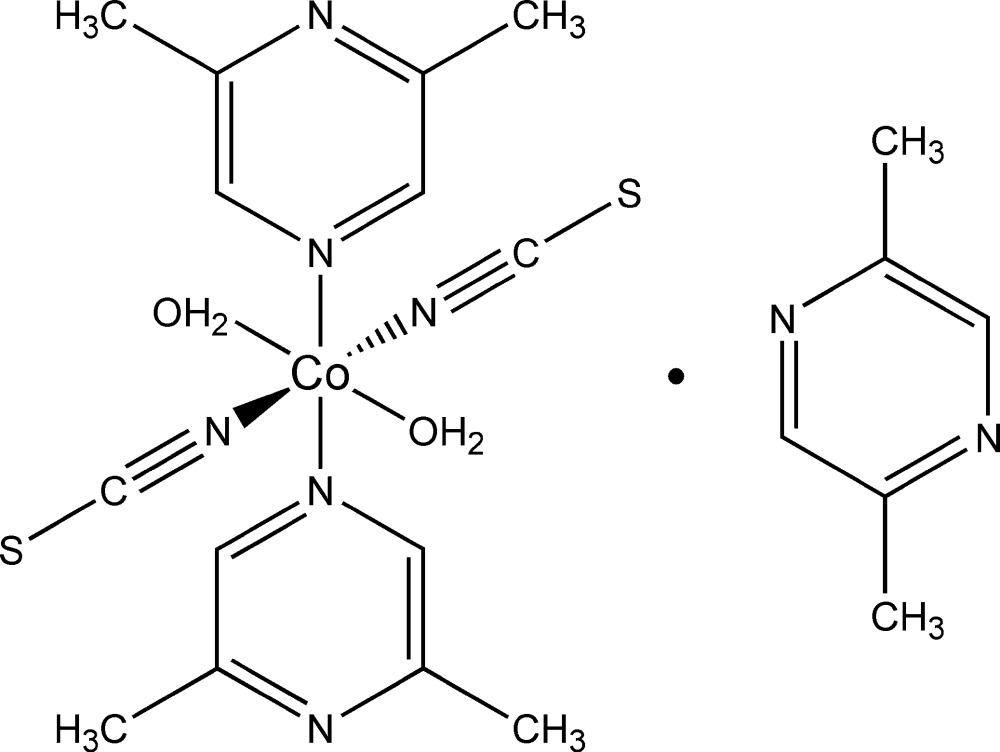


## Experimental   

### Crystal data  

[Co(NCS)_2_(C_6_H_8_N_2_)_2_(H_2_O)_2_]·C_6_H_8_N_2_
*M* *_r_* = 535.55
Triclinic, 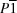

*a* = 8.3009 (4) Å
*b* = 9.0466 (5) Å
*c* = 10.4200 (6) Å
α = 96.640 (4)°
β = 105.820 (4)°
γ = 116.070 (4)°
*V* = 650.68 (7) Å^3^
*Z* = 1
Mo *K*α radiation
μ = 0.85 mm^−1^
*T* = 293 K
0.15 × 0.08 × 0.04 mm

### Data collection  

Stoe IPDS-2 diffractometer
Absorption correction: numerical (*X-SHAPE* and *X-RED32*; Stoe & Cie, 2008 ▸) *T*_min_ = 0.868, *T*_max_ = 0.959
10848 measured reflections
3447 independent reflections
3175 reflections with *I* > 2σ(*I*)
*R*_int_ = 0.031

### Refinement  

*R*[*F* ^2^ > 2σ(*F* ^2^)] = 0.035
*wR*(*F* ^2^) = 0.097
*S* = 1.05
3447 reflections
154 parameters
H-atom parameters constrained
Δρ_max_ = 0.43 e Å^−3^
Δρ_min_ = −0.39 e Å^−3^

### 

Data collection: *X-AREA* (Stoe & Cie, 2008 ▸); cell refinement: *X-AREA*; data reduction: *X-AREA*; program(s) used to solve structure: *SHELXS97* (Sheldrick, 2008 ▸); program(s) used to refine structure: *SHELXL2013* (Sheldrick, 2015 ▸); molecular graphics: *XP* in *SHELXTL* (Sheldrick, 2008 ▸) and *DIAMOND* (Brandenburg, 1999 ▸); software used to prepare material for publication: *publCIF* (Westrip, 2010 ▸).

## Supplementary Material

Crystal structure: contains datablock(s) I, global. DOI: 10.1107/S2056989015021829/wm5240sup1.cif

**Fig. 1.**
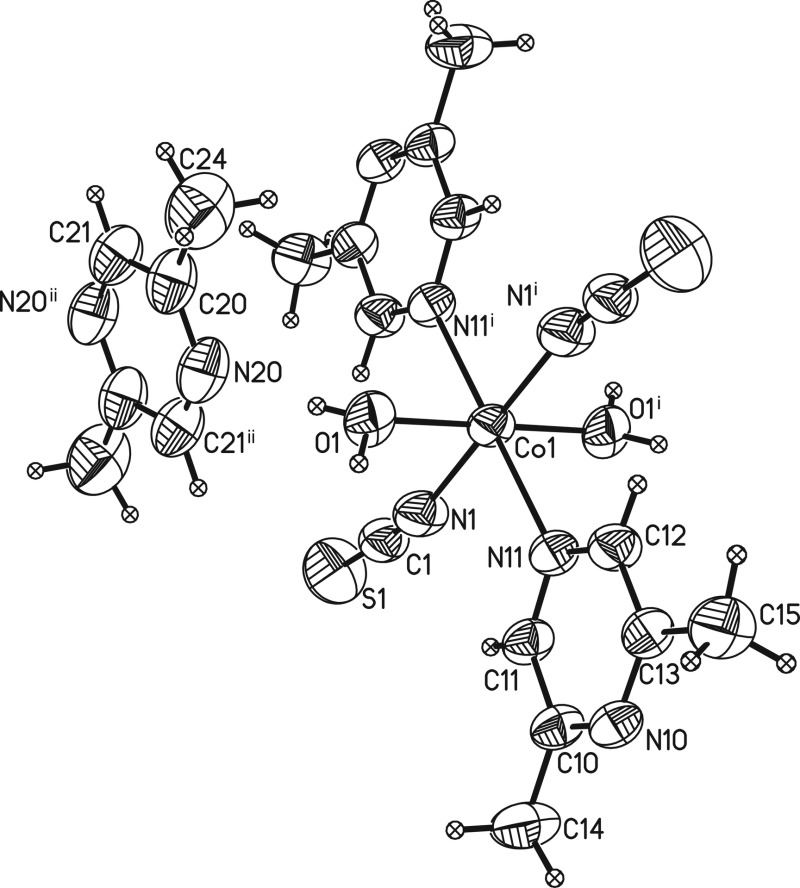
The molecular components in the crystal structure of the title compound. Displacement ellipsoids are drawn at the 50% probability level. [Symmetry codes: (i) -x + 1, y + 1, -z + 1; (ii) -x + 1, -y + 1, -z.]

Structure factors: contains datablock(s) I. DOI: 10.1107/S2056989015021829/wm5240Isup2.hkl

x y z x y z . DOI: 10.1107/S2056989015021829/wm5240fig1.tif
The mol­ecular components in the crystal structure of the title compound. Displacement ellipsoids are drawn at the 50% probability level. [Symmetry codes: (i) −*x* + 1, *y* + 1, −*z* + 1; (ii) −*x* + 1, −*y* + 1, −*z*.]

**Fig. 2.**
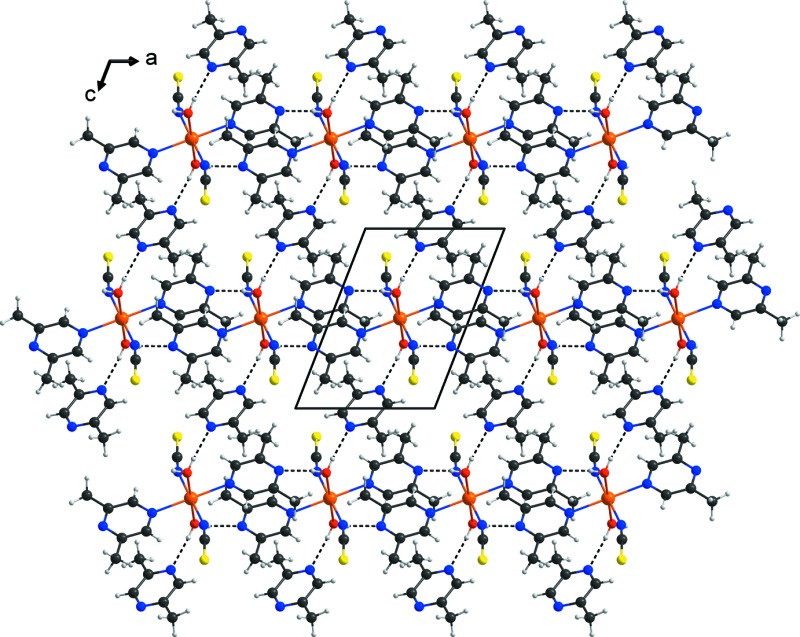
Crystal structure of the title compound in a view along [010]. O—H···N hydrogen bonding is shown as dashed lines.

. DOI: 10.1107/S2056989015021829/wm5240fig2.tif
Crystal structure of the title compound in a view along [010]. O—H⋯N hydrogen bonding is shown as dashed lines.

CCDC reference: 1437251

Additional supporting information:  crystallographic information; 3D view; checkCIF report

## Figures and Tables

**Table 1:** Hydrogen-bond geometry (Å, °)

*D*—H⋯*A*	*D*—H	H⋯*A*	*D*⋯*A*	*D*—H⋯*A*
O1—H2*O*1⋯N20	0.82	2.01	2.8193 (17)	172
O1—H1*O*1⋯N10^i^	0.82	2.01	2.8257 (15)	173

Symmetry code: (i) 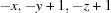.

## supplementary crystallographic information

### S1. Synthesis and crystallization

Co(SCN)_2_ and 2,5-di­methyl­pyrazine (97%) were purchased from Alfa Aesar. The title compound was prepared by the reaction of 57.9 mg (0.33 mmol) Co(SCN)_2_ and 140.0 µl 2,5-di­methyl­pyrazine (1.28 mmol) in 1.0 ml water at 393 K. After few days block-like crystals of the title compound were obtained that contained 2,6-di­methyl­pyrazine in addition. Later it was found that the commercially available 2,5-di­methyl­pyrazine contains about 3% of 2,6-di­methyl­pyrazine as a contamination.

### S2. Refinement

The carbon-bound H atoms were positioned with idealized geometry (methyl H atoms were allowed to rotate but not to tip) and were refined with *U*_iso_(H) = 1.2*U*_eq_(C) (1.5 for methyl H atoms) using a riding model with C—H = 0.93 Å for aromatic and C—H = 0.96 Å for methyl H atoms. The oxygen-bound H atoms were located in a difference map. The O—H bond length was constrained to 0.82 Å, with *U*_iso_(H) = 1.5*U*_eq_(O) using a riding model.

### Crystal data

**Table:**

[Co(NCS)_2_(C_6_H_8_N_2_)_2_(H_2_O)_2_]·C_6_H_8_N_2_	*Z* = 1
*M**_r_* = 535.55	*F*(000) = 279
Triclinic, *P*1	*D*_x_ = 1.367 Mg m^−^^3^
*a* = 8.3009 (4) Å	Mo *K*α radiation, λ = 0.71073 Å
*b* = 9.0466 (5) Å	Cell parameters from 10848 reflections
*c* = 10.4200 (6) Å	θ = 2.1–29.2°
α = 96.640 (4)°	µ = 0.85 mm^−^^1^
β = 105.820 (4)°	*T* = 293 K
γ = 116.070 (4)°	Block, purple
*V* = 650.68 (7) Å^3^	0.15 × 0.08 × 0.04 mm

### Data collection

**Table:**

Stoe IPDS-2 diffractometer	3175 reflections with *I* > 2σ(*I*)
Radiation source: fine-focus sealed tube	*R*_int_ = 0.031
ω scans	θ_max_ = 29.2°, θ_min_ = 2.9°
Absorption correction: numerical (*X-SHAPE* and *X-RED32*; Stoe & Cie, 2008)	*h* = −11→11
*T*_min_ = 0.868, *T*_max_ = 0.959	*k* = −12→12
10848 measured reflections	*l* = −14→14
3447 independent reflections	

### Refinement

**Table:**

Refinement on *F*^2^	0 restraints
Least-squares matrix: full	Hydrogen site location: mixed
*R*[*F*^2^ > 2σ(*F*^2^)] = 0.035	H-atom parameters constrained
*wR*(*F*^2^) = 0.097	*w* = 1/[σ^2^(*F*_o_^2^) + (0.0528*P*)^2^ + 0.1118*P*] where *P* = (*F*_o_^2^ + 2*F*_c_^2^)/3
*S* = 1.05	(Δ/σ)_max_ < 0.001
3447 reflections	Δρ_max_ = 0.43 e Å^−^^3^
154 parameters	Δρ_min_ = −0.39 e Å^−^^3^

### Special details

**Table:**

Geometry. All esds (except the esd in the dihedral angle between two l.s. planes) are estimated using the full covariance matrix. The cell esds are taken into account individually in the estimation of esds in distances, angles and torsion angles; correlations between esds in cell parameters are only used when they are defined by crystal symmetry. An approximate (isotropic) treatment of cell esds is used for estimating esds involving l.s. planes.

### Fractional atomic coordinates and isotropic or equivalent isotropic displacement parameters (Å^2^)

**Table:**

	*x*	*y*	*z*	*U*_iso_*/*U*_eq_	
Co1	0.5000	0.5000	0.5000	0.04299 (10)	
N1	0.3328 (2)	0.26365 (19)	0.35238 (16)	0.0590 (3)	
C1	0.2857 (2)	0.1399 (2)	0.27093 (18)	0.0558 (4)	
S1	0.23050 (11)	−0.02995 (8)	0.15745 (7)	0.0943 (2)	
N10	−0.05473 (17)	0.39022 (16)	0.65466 (14)	0.0467 (3)	
C10	−0.0663 (2)	0.27462 (19)	0.55572 (16)	0.0476 (3)	
C11	0.0930 (2)	0.30432 (19)	0.51972 (16)	0.0470 (3)	
H11	0.0815	0.2212	0.4508	0.056*	
C12	0.2717 (2)	0.5600 (2)	0.68289 (16)	0.0496 (3)	
H12	0.3881	0.6602	0.7301	0.059*	
C13	0.1150 (2)	0.5321 (2)	0.72101 (16)	0.0472 (3)	
C14	−0.2559 (3)	0.1149 (2)	0.4835 (2)	0.0718 (5)	
H14A	−0.3458	0.1151	0.5251	0.108*	
H14B	−0.2400	0.0171	0.4918	0.108*	
H14C	−0.3038	0.1098	0.3873	0.108*	
C15	0.1285 (3)	0.6597 (3)	0.8350 (2)	0.0706 (5)	
H15A	0.0349	0.6942	0.7993	0.106*	
H15B	0.2551	0.7579	0.8702	0.106*	
H15C	0.1043	0.6087	0.9083	0.106*	
N11	0.26138 (16)	0.44839 (16)	0.58104 (13)	0.0451 (3)	
N20	0.4442 (3)	0.5506 (2)	0.10190 (16)	0.0667 (4)	
C20	0.5941 (3)	0.6633 (3)	0.07748 (19)	0.0653 (4)	
C21	0.6487 (3)	0.6097 (3)	−0.0256 (2)	0.0676 (5)	
H21	0.7542	0.6894	−0.0414	0.081*	
C24	0.7003 (5)	0.8448 (3)	0.1628 (3)	0.1000 (9)	
H24A	0.6111	0.8861	0.1601	0.150*	
H24B	0.7935	0.9135	0.1263	0.150*	
H24C	0.7647	0.8517	0.2569	0.150*	
O1	0.40223 (16)	0.61584 (16)	0.35860 (12)	0.0560 (3)	
H1O1	0.2978	0.6105	0.3470	0.084*	
H2O1	0.4074	0.6011	0.2810	0.084*	

### Atomic displacement parameters (Å^2^)

**Table:**

	*U*^11^	*U*^22^	*U*^33^	*U*^12^	*U*^13^	*U*^23^
Co1	0.03641 (14)	0.05128 (17)	0.04350 (15)	0.02088 (11)	0.02181 (10)	0.00580 (10)
N1	0.0497 (7)	0.0613 (8)	0.0578 (8)	0.0190 (6)	0.0283 (6)	0.0010 (6)
C1	0.0474 (7)	0.0589 (9)	0.0522 (8)	0.0181 (6)	0.0230 (6)	0.0064 (7)
S1	0.1115 (5)	0.0684 (3)	0.0760 (4)	0.0327 (3)	0.0290 (3)	−0.0124 (3)
N10	0.0437 (6)	0.0543 (7)	0.0542 (7)	0.0274 (5)	0.0288 (5)	0.0168 (5)
C10	0.0441 (6)	0.0505 (7)	0.0552 (8)	0.0230 (6)	0.0282 (6)	0.0152 (6)
C11	0.0457 (7)	0.0508 (7)	0.0521 (7)	0.0248 (6)	0.0279 (6)	0.0115 (6)
C12	0.0398 (6)	0.0572 (8)	0.0516 (7)	0.0225 (6)	0.0214 (6)	0.0083 (6)
C13	0.0462 (7)	0.0567 (8)	0.0486 (7)	0.0293 (6)	0.0250 (6)	0.0123 (6)
C14	0.0537 (9)	0.0600 (10)	0.0881 (14)	0.0129 (8)	0.0396 (9)	0.0031 (9)
C15	0.0662 (10)	0.0745 (12)	0.0702 (11)	0.0334 (9)	0.0348 (9)	−0.0027 (9)
N11	0.0396 (5)	0.0555 (7)	0.0479 (6)	0.0256 (5)	0.0235 (5)	0.0131 (5)
N20	0.0921 (11)	0.0828 (10)	0.0545 (8)	0.0558 (9)	0.0451 (8)	0.0220 (7)
C20	0.0919 (13)	0.0714 (11)	0.0511 (9)	0.0488 (10)	0.0364 (9)	0.0185 (8)
C21	0.0838 (12)	0.0782 (12)	0.0593 (10)	0.0441 (10)	0.0434 (9)	0.0228 (9)
C24	0.141 (2)	0.0768 (15)	0.0808 (16)	0.0478 (16)	0.0543 (17)	0.0092 (12)
O1	0.0550 (6)	0.0824 (8)	0.0515 (6)	0.0436 (6)	0.0317 (5)	0.0194 (5)

### Geometric parameters (Å, º)

**Table:**

Co1—N1^i^	2.0812 (15)	C14—H14A	0.9600
Co1—N1	2.0812 (15)	C14—H14B	0.9600
Co1—O1	2.0930 (12)	C14—H14C	0.9600
Co1—O1^i^	2.0930 (12)	C15—H15A	0.9600
Co1—N11	2.2460 (11)	C15—H15B	0.9600
Co1—N11^i^	2.2460 (11)	C15—H15C	0.9600
N1—C1	1.158 (2)	N20—C21^ii^	1.322 (3)
C1—S1	1.6232 (18)	N20—C20	1.330 (3)
N10—C10	1.3321 (19)	C20—C21	1.389 (2)
N10—C13	1.336 (2)	C20—C24	1.493 (3)
C10—C11	1.3935 (18)	C21—N20^ii^	1.322 (3)
C10—C14	1.495 (2)	C21—H21	0.9300
C11—N11	1.3343 (18)	C24—H24A	0.9600
C11—H11	0.9300	C24—H24B	0.9600
C12—N11	1.3342 (18)	C24—H24C	0.9600
C12—C13	1.3893 (18)	O1—H1O1	0.8201
C12—H12	0.9300	O1—H2O1	0.8200
C13—C15	1.501 (2)		
			
N1^i^—Co1—N1	180.00 (9)	C10—C14—H14B	109.5
N1^i^—Co1—O1	89.38 (6)	H14A—C14—H14B	109.5
N1—Co1—O1	90.62 (6)	C10—C14—H14C	109.5
N1^i^—Co1—O1^i^	90.62 (6)	H14A—C14—H14C	109.5
N1—Co1—O1^i^	89.38 (6)	H14B—C14—H14C	109.5
O1—Co1—O1^i^	180.0	C13—C15—H15A	109.5
N1^i^—Co1—N11	88.99 (5)	C13—C15—H15B	109.5
N1—Co1—N11	91.01 (5)	H15A—C15—H15B	109.5
O1—Co1—N11	92.08 (4)	C13—C15—H15C	109.5
O1^i^—Co1—N11	87.92 (4)	H15A—C15—H15C	109.5
N1^i^—Co1—N11^i^	91.01 (5)	H15B—C15—H15C	109.5
N1—Co1—N11^i^	88.99 (5)	C12—N11—C11	116.38 (12)
O1—Co1—N11^i^	87.92 (4)	C12—N11—Co1	123.72 (10)
O1^i^—Co1—N11^i^	92.08 (4)	C11—N11—Co1	119.73 (9)
N11—Co1—N11^i^	180.0	C21^ii^—N20—C20	118.15 (15)
C1—N1—Co1	162.42 (13)	N20—C20—C21	119.60 (19)
N1—C1—S1	177.21 (15)	N20—C20—C24	118.75 (18)
C10—N10—C13	118.09 (12)	C21—C20—C24	121.6 (2)
N10—C10—C11	120.65 (14)	N20^ii^—C21—C20	122.25 (19)
N10—C10—C14	117.78 (13)	N20^ii^—C21—H21	118.9
C11—C10—C14	121.56 (15)	C20—C21—H21	118.9
N11—C11—C10	122.01 (13)	C20—C24—H24A	109.5
N11—C11—H11	119.0	C20—C24—H24B	109.5
C10—C11—H11	119.0	H24A—C24—H24B	109.5
N11—C12—C13	122.41 (14)	C20—C24—H24C	109.5
N11—C12—H12	118.8	H24A—C24—H24C	109.5
C13—C12—H12	118.8	H24B—C24—H24C	109.5
N10—C13—C12	120.37 (14)	Co1—O1—H1O1	119.4
N10—C13—C15	117.87 (13)	Co1—O1—H2O1	120.2
C12—C13—C15	121.76 (15)	H1O1—O1—H2O1	105.4
C10—C14—H14A	109.5		

Symmetry codes: (i) −*x*+1, −*y*+1, −*z*+1; (ii) −*x*+1, −*y*+1, −*z*.

### Hydrogen-bond geometry (Å, º)

**Table:**

*D*—H···*A*	*D*—H	H···*A*	*D*···*A*	*D*—H···*A*
O1—H2*O*1···N20	0.82	2.01	2.8193 (17)	172
O1—H1*O*1···N10^iii^	0.82	2.01	2.8257 (15)	173

Symmetry code: (iii) −*x*, −*y*+1, −*z*+1.
