# Supplementary figures and images for: Crystal structure of di­aqua­bis­(2,6-di­methyl­pyrazine-κN 4)bis­(thio­cyanato-κN)cobalt(II) 2,5-di­methyl­pyrazine monosolvate
Source: Acta Crystallogr E Crystallogr Commun. 2015 Dec 6;71(Pt 12):m242–3. doi: 10.1107/S2056989015021829 (PMC4719854; doi:10.1107/S2056989015021829)

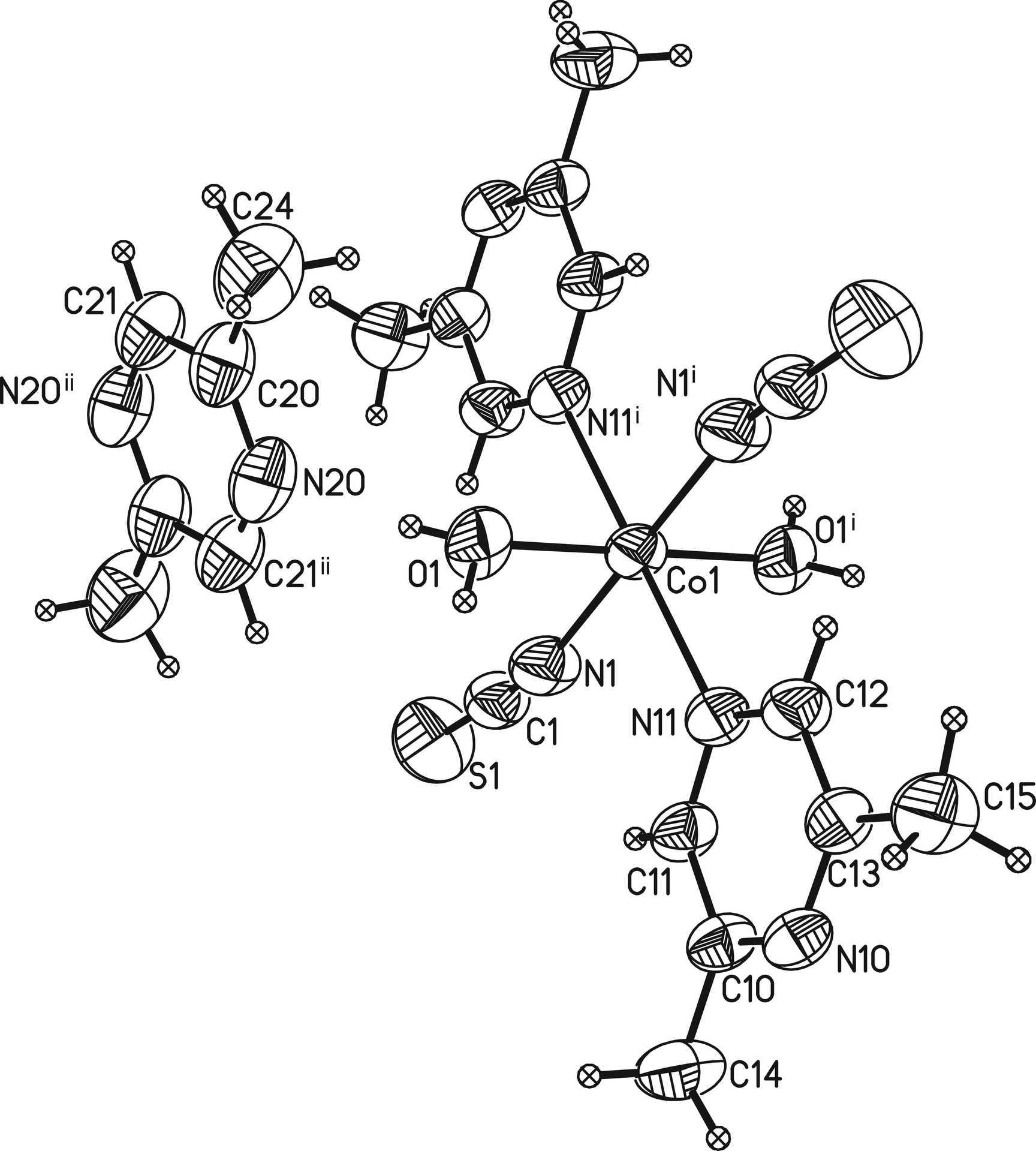

Supplement: Supplementary file 3 [file e-71-0m242-fig1.tif]

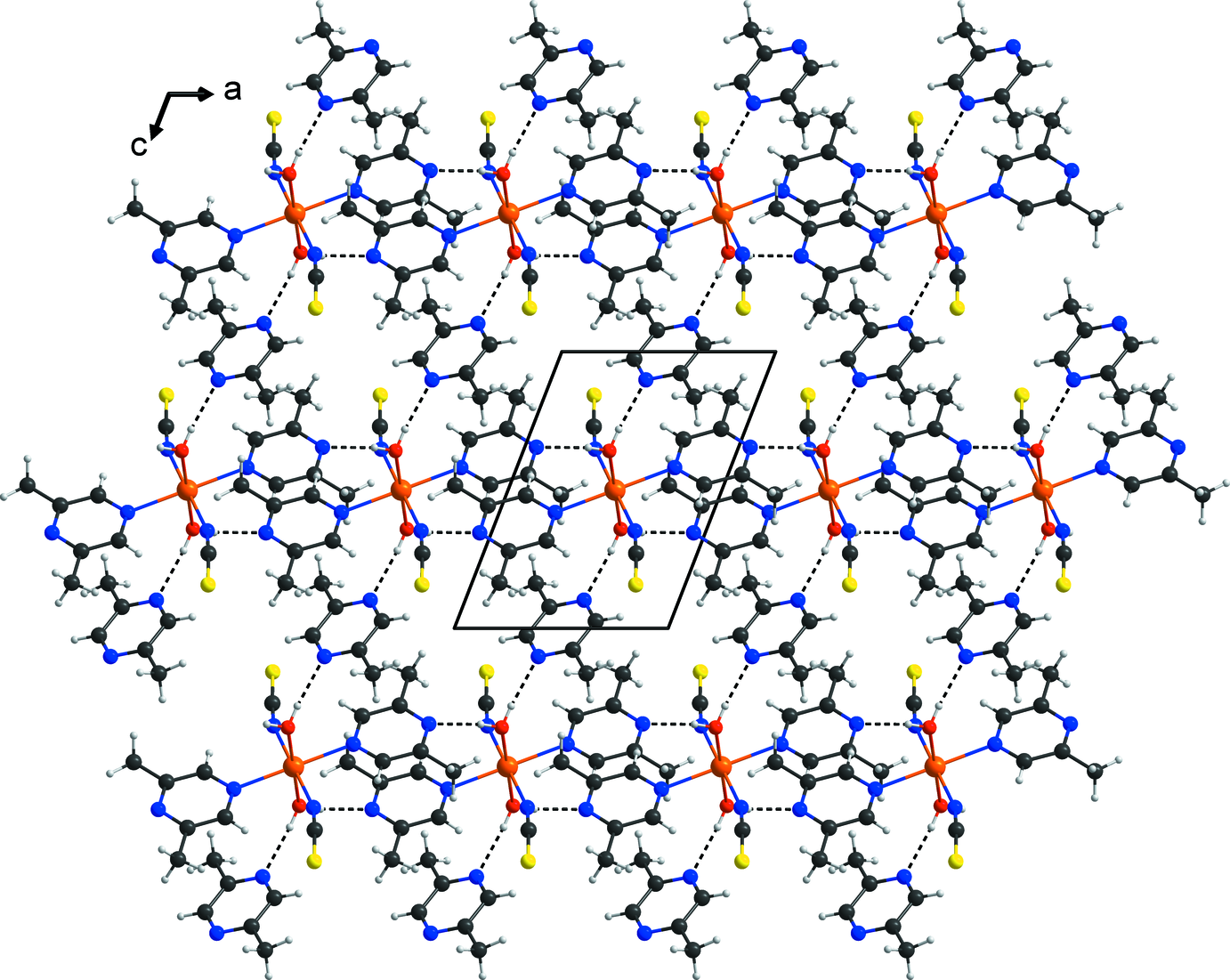

Supplement: Supplementary file 4 [file e-71-0m242-fig2.tif]
